# Pyocyanin Restricts Social Cheating in *Pseudomonas aeruginosa*

**DOI:** 10.3389/fmicb.2018.01348

**Published:** 2018-06-27

**Authors:** Paulina Castañeda-Tamez, Jimena Ramírez-Peris, Judith Pérez-Velázquez, Christina Kuttler, Ammar Jalalimanesh, Miguel Á. Saucedo-Mora, J. Guillermo Jiménez-Cortés, Toshinari Maeda, Yael González, María Tomás, Thomas K. Wood, Rodolfo García-Contreras

**Affiliations:** ^1^Department of Microbiology and Parasitology, Faculty of Medicine, Universidad Nacional Autónoma de México, Mexico City, Mexico; ^2^Instituto Nacional de Psiquiatría Ramón de la Fuente Muñiz, Mexico City, Mexico; ^3^Institute of Computational Biology, Helmholtz Zentrum München, Deutsches Forschungszentrum für Gesundheit und Umwelt (GmbH), Neuherberg, Germany; ^4^Zentrum Mathematik, Technische Universität München, Munich, Germany; ^5^Iranian Research Institute for Information Science and Technology (IRANDOC), Tehran, Iran; ^6^Department of Biological Functions Engineering, Kyushu Institute of Technology, Kitakyushu, Japan; ^7^Department of Microbiology, Instituto de Investigación Biomédica de A Coruña, Complexo Hospitalario Universitario de A Coruña, SERGAS, Universidade da Coruña, A Coruña, Spain; ^8^Department of Chemical Engineering, The Pennsylvania State University, State College, PA, United States

**Keywords:** public goods, pyocyanin, social cheating, quorum sensing (QS), oxidative stress, policing

## Abstract

Quorum sensing (QS) in *Pseudomonas aeruginosa* coordinates the expression of virulence factors, such as exoproteases and siderophores, that are public goods utilized by the whole population of bacteria, regardless of whether they invested or not in their production. These public goods can be used by QS defective mutants for growth, and since these mutants do not contribute to public goods production, they are considered social cheaters. Pyocyanin is a phenazine that is a toxic, QS-controlled metabolite produced by *P. aeruginosa*. It is a redox-active compound and promotes the generation of reactive oxygen species; it also possesses antibacterial properties and increases fitness in competition with other bacterial species. Since QS-deficient individuals are less able to tolerate oxidative stress, we hypothesized that the pyocyanin produced by the wild-type population could promote selection of functional QS systems in this bacterium. Here, we demonstrate, using competition experiments and mathematical models, that, indeed, pyocyanin increases the fitness of the cooperative QS-proficient individuals and restricts the appearance of social cheaters. In addition, we also show that pyocyanin is able to select QS in other bacteria such as *Acinetobacter baumannii*.

## Introduction

*Pseudomonas aeruginosa* uses QS to estimate its population density and to reprogram its gene expression and behavior accordingly. This system relies on the continuous production of small chemical signals known as autoinducers which are produced at a low rate until they accumulate and interact with their receptors. The receptors are activated by signal binding and induce transcription of the promoters of the genes encoding the enzymes that synthesize the autoinducers, which results in a sudden increase in autoinducer concentrations and receptor activation. Receptor activation also allows for increased transcription of several other genes allowing the coordination of the expression of cooperative behaviors like the production of costly exoproducts such as exoenzymes and siderophores (Popat et al., 2015; García-Contreras, 2016). In *P. aeruginosa*, at least four interconnected QS systems exist; for two of them, LasIR and RhlIR, the autoinducer signals are *N*-acyl homoserine lactones while for the third system, PQS, hydrophobic quinolones are used, and for the fourth system, IQS, which is active mainly under conditions of phosphate starvation, 2-(2-hydroxyphenyl)-thiazole-4-carbaldehyde is used as the signal (Lee and Zhang, 2015). These subsystems are hierarchically organized, with LasIR at the top, in charge of the activation of the other systems (Castillo-Juarez et al., 2015; Papenfort and Bassler, 2016). In general, the costly exoproduct production which QS regulates can benefit all the individuals in the population, regardless if they invested in their production or not; hence, these exoproducts are public goods. Since the production of public goods is costly, the individuals that use them without contributing to their production are social cheaters (Diggle et al., 2007) and can invade the population, causing a tragedy of the commons (Sandoz et al., 2007). It has been demonstrated in *P. aeruginosa* that cheating behavior exists for both exoprotease production (Diggle et al., 2007) and for the production of the main siderophore pyoverdine (Kummerli et al., 2009). In the first case, cheaters (mutants deficient in *lasR*) naturally emerge in cultures with protein as the sole carbon source and also have been isolated from several environments such as the lungs of cystic fibrosis patients (Sandoz et al., 2007; Hoffman et al., 2009; Dandekar et al., 2012; García-Contreras et al., 2015a). In the second case, mutants unable to produce pyoverdine are selected in iron deficient media (Dumas and Kummerli, 2012).

However, although cheating exists in nature, QS systems that regulate public goods are common; hence, factors that counteract the effects of social cheaters should exist. For *P. aeruginosa*, those mechanisms include the growth of bacteria in conditions that allow the physical separation of cooperators and cheaters, decreasing the cheater’s fitness (Hense et al., 2007; Mund et al., 2016). This occurs during the growth in highly viscous medium, since viscosity limits diffusion of public goods such as siderophores (Kummerli et al., 2009). Another way to counteract social cheating is to combine the assimilation of protein, which depends on the production of exoproteases that are exploited by cheaters, with the assimilation of adenosine, whose catabolism is also QS-dependent, but in contrast to the extracellular protein, it is mediated by intracellular private enzymes (Dandekar et al., 2013). Finally environmental factors such as the presence of toxic compounds such as H_2_O_2_ (García-Contreras et al., 2015b) and HCN (Wang et al., 2015), and the presence of some temperate phages (Saucedo-Mora et al., 2017) also restrict social cheating, since QS deficient mutants are more sensitive to stress due a lower expression of anti-oxidant enzymes such as catalase and superoxide dismutase (Hassett et al., 1999), and likely due to a lack of modifications that enhance membrane tolerance to stress (Davenport et al., 2015). In addition, a lower expression of cyanide insensitive cytochrome oxidase may contribute to their higher sensitivity toward HCN (Wang et al., 2015).

One of the main QS-controlled virulence factors are the phenazines, dibenzo annulated pyrazines with diverse activities such as: (i) cell signaling (Dietrich et al., 2006), (ii) biofilm formation (Ramos et al., 2010; Wang et al., 2011), (iii) survival in anaerobiosis (Wang et al., 2010), (iv) fitness in the presence of competitor bacteria (Smalley et al., 2015), (v) resistance to gallium nitrate (García-Contreras et al., 2013; Rezzoagli et al., 2017) and other toxic metals such as silver (Muller and Merrett, 2014), and (vi), damage to host cells (Rada and Leto, 2013), among others.

Pyocyanin, one of the main phenazines of *P. aeruginosa*, is produced by several clinical strains from pulmonary and extra-pulmonary infections (Schaber et al., 2004; García-Contreras et al., 2015b; Guendouze et al., 2017) as well as in environmental strains (Grosso-Becerra et al., 2014) and is found in high concentrations [up to 100 μM (Caldwell et al., 2009) in the lung of cystic fibrosis patients]. Due to its redox activity, pyocyanin increases the production of reactive oxygen species by donating electrons to oxygen, thereby producing hydrogen peroxide. Moreover, pyocyanin also depletes the pools of antioxidant molecules like glutathione. Taking these results into account as well as the fact that QS-defective mutants are less able to cope with oxidative stress (García-Contreras et al., 2015b) and likely have membranes more sensitive to oxidative stress than QS-proficient individuals due lower levels of cyclopropanation and lower levels of fatty acid saturation (Davenport et al., 2015), we hypothesized that pyocyanin may differentially affect this population and may act as a policing metabolite. To test this hypothesis, we performed competition experiments and developed mathematical models; our results show that indeed the production of this metabolite selects for the presence of QS.

## Materials and Methods

### Bacterial Strains and Growth Conditions

*Pseudomonas aeruginosa* PA14, and the *lasR rhlR* mutant were donated by Dr. You-Hee Cho from the College of Pharmacy, CHA University, South Korea (Park et al., 2005). The PA14 *phzM* mutant (40343 from the collection) was provided by Dr. Frederick Ausubel from the Harvard Medical School (Liberati et al., 2006). In order to avoid differences between PA14 genetic backgrounds, the *phzM* mutation was incorporated into the PA14 strain from South Korea by phage transfection using the DMS3 phage (Budzik et al., 2004). The *Acinetobacter baumannii* mutant with the *abaI* gene deleted was generated using the pMo130 plasmid (Hamad et al., 2009) which carries the *aphA* gene that provides kanamycin resistance, the *xylE* gene that allows visual detection of the mutants, and the modified *sacB* gene that allows the resolution of the co-integrants. The mutants were confirmed by sequencing and real-time PCR with Taqman probes (see Supplementary Table S1 for the primer sequences).

Pre-cultures of all strains were grown in LB at 37°C with 200 rpm shaking for ∼16 h. For the *phzM* mutant, gentamicin at 15 μg mL^−1^ was added. The pre-cultures were then used for the inoculation of flasks with M9 minimal medium supplemented with 0.25% of sodium caseinate as the sole carbon source (M9 caseinate medium) with or without exogenous protease (type XIV p5147, from SIGMA at 1 unit/ml) or with the same medium with 0.25% of casamino acids as a sole carbon source or with LB medium, and the cultures were grown under the same conditions. Growth was monitored by recording the increase of the turbidity (600 nm) with a spectrophotometer (UV-1800, Shimadzu).

### Bactericidal Effect of Pyocyanin

To evaluate the bactericidal effect of pyocyanin, wild-type, the *lasR rhlR* mutant and the *phzM* mutant cells were cultured in LB to turbidity at 600 nm of 1.0, then samples (1 mL) were taken and pyocyanin at 50 or 100 μM was added. Cells were exposed for 30 min, and viable counts were used to determine the degree of survival.

### Effect of Pyocyanin in the Selection of Social Cheaters

Single colonies were inoculated into LB medium to form precultures that were transferred to M9 caseinate medium (initial turbidity of ∼0.05 at 600 nm); each culture was incubated for 24 h and was used for inoculating subsequent cultures (also at an initial turbidity of ∼0.05) with or without the addition of 100 μM pyocyanin (added to the medium before inoculation). The percentage of protease-less producer colonies after each 24 h culture was evaluated by plating colonies (between 25 and 50 per culture) and visualizing the casein degradation halo in LB plates with 3% skimmed milk.

### Competition Experiments

Cultures in M9 caseinate medium were inoculated with different proportions of the wild-type PA14 strain and the *lasR rhlR* mutant or the *phzM* mutant and the *lasR rhlR* mutant. For all cases, enough bacteria to achieve an initial turbidity at 600 nm of ∼0.05 was used, then the cultures were grown under the same conditions detailed above. Samples of each culture were taken after 0, 4, 6, 10, and 24 h of cultivation and were used to isolate colonies. The proportion of PA14 and the *lasR rhlR* mutant was determined by evaluating casein degradation.

For evaluating the effect of pyocyanin in the competition between the *lasR rhlR* mutant and the *phzM* mutant, pyocyanin was purified by the extraction with chloroform and 0.2 M HCl (Essar et al., 1990), and its purity was verified by comparing its absorbance spectrum with commercial pyocyanin (SIGMA, St. Louis, MO, United States). Pyocyanin (neutralized with NaOH) was added at 25 μM to the cultures, and at 4, 6, 10, and 24 h, samples were taken to estimate the proportion of QS mutant and *phzM* populations.

### *A. baumannii* Competition

*Acinetobacter baumannii* ATCC-17978 and its isogenic *abaI* mutant were cultured in LB medium until they reached to the early stationary phase (turbidity at 600 nm ∼2.0), and then 500 μl of each culture was mixed in 2 mL tubes with or without 50 μM of pyocyanin and incubated for 20 min under the same conditions. Samples of the mixtures were taken before adding pyocyanin and after the incubations for isolating colonies that were then plated on blood agar plates; plates were incubated for 16 h at 37°C and then the identity of these colonies was determined by PCR. A total of 140 colonies were screened using two PCR reactions: one for the identification of the *abaI* gene and the other for the house keeping gene *pbpC* using primers 5′-CCAATATCATTGGTTGTGCC and 5′-TCGTAATGAGTTGTTTTGCG that yield a product of 255 bp for *abaI*, and the primers 5′-TTTGACTGGGATGGTATTCG and 5′-GGAATCTGGGTGCTATTCAT that yield a product of 419 bp for *pbpC*.

### Pyocyanin and Exoprotease Assay

Pyocyanin concentrations were determined spectrophotometrically after the extraction with chloroform and 0.2 M HCl (Essar et al., 1990). The absorbance at 520 nm was used with an 𝜀 of 2.46 mM^−1^ cm^−1^ (O’Malley et al., 2004). Elastase was determined by quantifying the production of Congo red from the hydrolysis elastine-Congo red complex (SIGMA) (Ohman et al., 1980), and alkaline protease concentrations were determined by the production of remazol brilliant blue R from the hydrolysis of the Hide–Remazol Brilliant Blue R powder (SIGMA) (Howe and Iglewski, 1984).

### Catalase Assay

Catalase activity was determined by recording the conversion of H_2_O_2_ to H_2_O + O_2_, spectrophotometrically at 240 nm and 37°C in Tris (50 mM at pH 7). PA14 and the QS mutants cells were grown in M9 medium with 0.25% of casamino acids to the middle exponential phase (turbidity ∼1.0 at 600 nm), then 1 mL of each culture was harvested by centrifugation; the cell pellets were washed in the same reaction buffer once, resuspended in 250 μL of the buffer, and half of the cells were mixed with 125 μL of a permeabilization solution (100 mM dibasic sodium phosphate, 20 mM KCl, 2 mM MgSO_4_, 0.8 mg/mL CTAB, 0.4 mg/mL sodium deoxycholate), incubated for 20 min at 37°C, and then used to determine activity. In parallel, 1 mL of each culture was exposed to 25 μM of pyocyanin at 37°C for 30 min and after that, cell pellets were obtained and the procedures described before were followed to determine the effect of pyocyanin in the catalase activity. Protein concentrations were determined by the Bradford method, and catalase activity was expressed as nanomol of H_2_O_2_ degraded per minute per mg of protein.

### Statistical Analysis

All experiments were done at least in triplicate. Statistical significances for **Figures 3, 4, 6** were evaluated by using a Student’s two-tailed test and considered significant if *P* < 0.05. Statistical significance for **Figure 5** was evaluated by a chi-squared test and considered significant if *P* < 0.05.

### Mathematical Modeling

A spatial stochastic mathematical model was developed to further explore the role of pyocyanin in the selection of the QS phenotype during the competition experiments. A deterministic mathematical model was used to compute some of the parameters involved. A detailed description of the models can be found in the Supplementary Materials.

## Results

### Pyocyanin Selects the Wild-Type Phenotype in Competition With Non-protease Producer Mutants

Our first approximation to evaluate factors that stabilize QS in *P. aeruginosa* was to test their effect in the outcome of competition experiments between the PA14 wild-type strain and non-protease producer, the *lasR rhlR* mutant, in medium with caseinate as the sole carbon source. In this medium, the only way mutants can grow is by utilizing the peptides and amino acids produced by the hydrolysis of casein by the QS-controlled exoproteases synthetized and exported by the wild-type strain; hence, *lasR rhlR* mutants behaved as social cheaters that exploit the cooperator (wild-type) individuals (Diggle et al., 2007; García-Contreras et al., 2015b). Growth curves of PA14, the *lasR rhlR* mutant, and their mixtures containing an initial proportion of 10, 50, and 90% of the *lasR rhlR* mutant population confirmed that the *lasR rhlR* mutant was unable to grow in caseinate as the sole carbon source after 24 h of cultivation and show that increasing the initial proportion of the *lasR rhlR* mutant decreased the growth yields of the cultures after 24 h of cultivation (Supplementary Figure S1). In addition, the overall growth rate of the population also decreased with increasing initial amounts of the *lasR rhlR* mutant, indicating that the presence of QS cheaters is a burden for the system’s growth.

**FIGURE 1:**
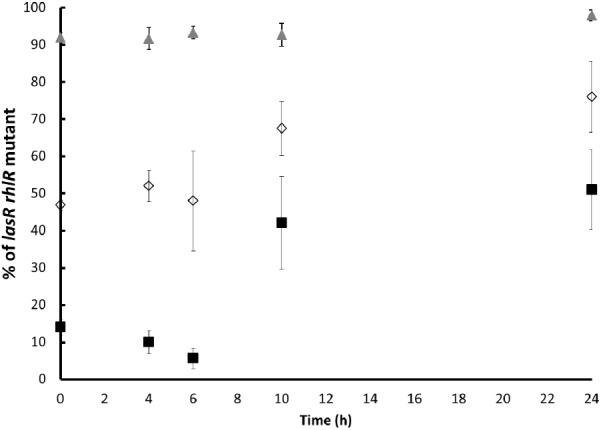
Proportion of the *lasR rhlR* mutant during competition with the PA14 wild-type in caseinate medium. The initial proportion of the mutant was approximately 10% (black squares), 50% (white diamonds), and 90% (gray triangles). Experiments were performed in quintuplicate, and the average ± SEM are shown. The differences between all the proportions at the initial time vs. all the proportions at 10 h are significant, except for the proportion of 90%. The differences between all the proportions at the initial time vs. all the proportions at 24 h were significant, and no significant difference was found between 10 and 24 h for any case (*P* < 0.05 in a one tailed *T*-student test).

In agreement with previous findings, the proportion of the *lasR rhlR* mutant populations substantially increased with time (Mellbye and Schuster, 2011; Gerdt and Blackwell, 2014). After 24 h of culture, the percentage of the *lasR rhlR* mutant was of approximately 50, 75, and 97% when the initial proportions were approximately 10, 50, and 90%, respectively (**Figure 1**), validating that the *lasR rhlR* mutant grows at the expense of the wild-type that produced exoprotease under this condition.

**FIGURE 2:**
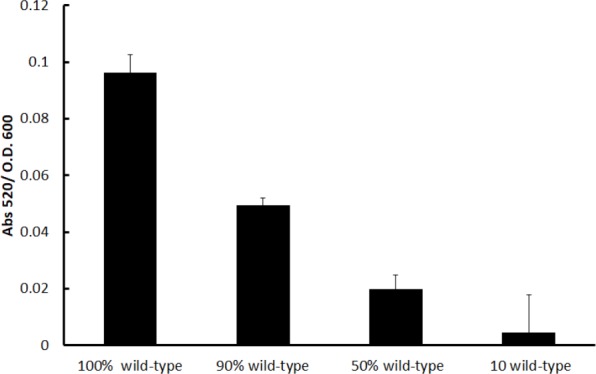
Normalized pyocyanin production in the competition cultures after 24 h. The percentage indicated in the abscissa corresponds to the initial wild-type population (the remainder is the proportion of the *lasR rhlR* mutant). Experiments were done in triplicate, and the average ± SEM are shown.

These equilibrium final proportions were reached around 10 h and remained mostly stable (the mutant fraction only increased slightly) after 24 h. Critically, the stable final proportions consisted of lower amounts of the mutant when the amount of mutants in the inoculum was lower; hence, the final proportions depended on the initial proportions, indicating that probably factors produced by the cooperative PA14 individuals were restricting further increases in the mutant proportion. In our experiments, it was clear that the production of a potential toxic metabolite by the wild-type PA14 population, the phenazine pyocyanin, was high and its amount positively correlated with a higher initial wild-type proportion as expected (**Figure 2**). The production of pyocyanin is positively regulated by the LasR/RhlR systems (Dekimpe and Deziel, 2009), and the mutant we used was unable to produce pyocyanin (García-Contreras et al., 2015b).

**FIGURE 3:**
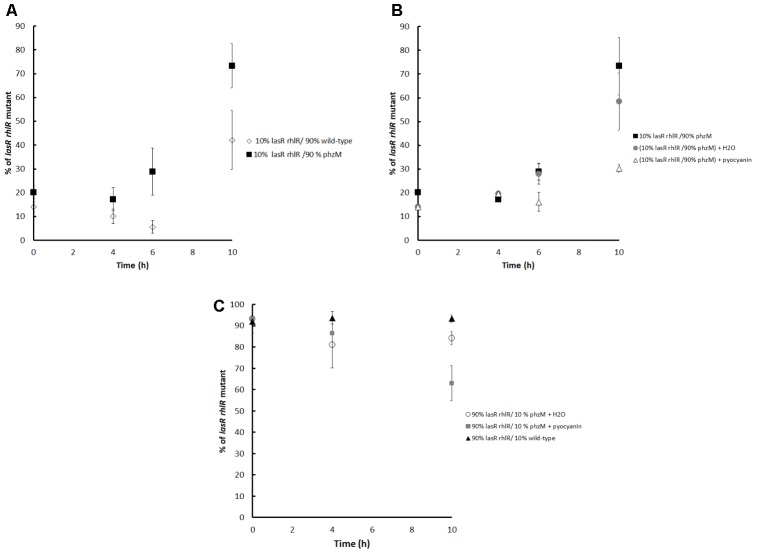
**(A)** Competitions between the *lasR rhlR* mutant (cheater) and the *phzM* mutant (unable to make pyocyanin) grown in M9 caseinate medium. For comparison, competition between the PA14 wild-type and the *lasR rhlR* mutant is also shown. The initial proportion of the *lasR rhlR* mutant was ∼10%. **(B)** Effect of adding pyocyanin (50 μM at 4 h) to the competition experiments between the *lasR rhlR* mutant and the *phzM* mutant. The negative controls are H_2_O or no compound added, and the initial percentage of the *lasR rhlR* mutant was ∼10%. **(C)** Competitions between the PA14 wild-type and the *lasR rhlR* mutant and between the *phzM* and *lasR rhlR* mutants with and without pyocyanin when the initial proportion of the *lasR rhlR* mutant was ∼90%. Average of 4 independent cultures ± SEM are shown. For **(A)**, differences between the proportions of both curves at 6 and 10 h are statistically significant (*P* < 0.05 in a two tailed *T*-student test). For **(B)**, differences between the control curve and the curve with added H_2_O are not significant, but they are statistically significant when compared to the curve with added pyocyanin at 6 and 10 h. For **(C)**, the differences between the three curves are significant at 10 h (*P* < 0.05 in a two tailed *T*-student test).

Therefore, competition experiments between a non-pyocyanin producer but QS-active strain, the *phzM* mutant, and the *lasR rhlR* mutant were conducted; the results indicated that after 6 and 10 h, the relative frequency of the *lasR rhlR* mutant was significantly higher than when they were competing against PA14 when the initial mutant proportion was 10% (**Figure 3A**). In agreement, the addition of pyocyanin to the *phzM* vs. *lasR rhlR* mutant competition experiments strongly decreased the frequency of the *lasR rhlR* mutant at 10 h for initial proportions of 10% (**Figure 3B**) and 90% of mutants (**Figure 3C**), demonstrating that in addition to HCN, pyocyanin was also capable of restricting cheating (*lasR rhlR* mutant).

Control growth experiments of the three strains (PA14 wild-type, the *lasR rhlR* mutant, and the *phzM* mutant) in M9 medium with 0.25% of casamino acids as the sole carbon source and with 0.25% caseinate plus exogenous protease showed almost no difference in the growth rates of the strains **Table 1** and Supplementary Figures S2A,B.

**Table 1:** *P. aeruginosa* strains characteristics.

Strain	Growth rate in caseinate 0.25% (h^−1^)	Growth rate in caseinate 0.25% +protease (h^−1^)	Growth rate in casamino acids 0.25% (h^−1^)	Pyocyanin (μM)	Exoprotease skimmed milk	Elastase (Abs 595 nm)	Collagenase (Abs 495 nm)
PA14 wt	0.39 ± 0.12	0.73 ± 0.05	1.06 ± 0.02	127 ± 0.8	++	3.36 ± 0.49	2.4 ± 0.42
*phzM*	0.12 ± 0.02	0.73 ± 0.04	0.99 ± 0.04	1 ± 0.3	+	1.18 ± 0.24	1.06 ± 0.9
*lasR rhlR*	Do not growth	0.79 ± 0.03	1.07 ± 0.026	Not determined	−	Not determined	Not determined

### Pyocyanin Toxicity

**FIGURE 4:**
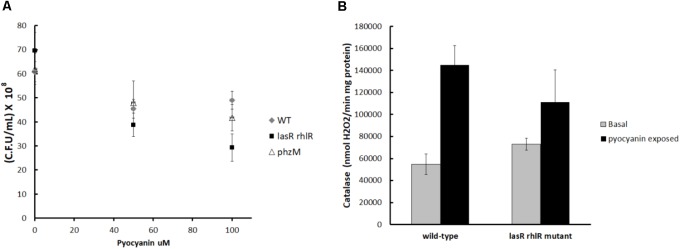
**(A)** Bactericidal effect of pyocyanin on PA14, the *lasR rhlR* mutant, and the *phzM* mutant. Cells were cultured in LB to turbidity at 600 nm of 1.0, then samples (1 mL) were taken and pyocyanin was added at the indicated concentrations. Cells were exposed 30 min, and then viable counts were used to determine the degree of survival. Experiments were done in quadruplicate, and the average ± SEM is shown. The difference between the survival of the wild-type strain and the *lasR rhlR* mutant with 100 ≠M of pyocyanin is significant (*P* < 0.05 in a two tailed *T*-student test). **(B)** Effect of pyocyanin on the catalase activity of PA14 and the *lasR rhlR* mutant. The difference between the catalase activity of the wild-type with and without pyocyanin is significant (*P* < 0.05 in a two tailed *T*-student test).

In agreement with the competition experiments, the bactericidal effect of pyocyanin on the *lasR rhlR* mutant was greater than its effect on PA14 and the *phzM* mutant (**Figure 4A**). Also the addition of pyocyanin was able to significantly induce the activity of catalase in PA14 cultures but not in the *lasR rhlR* mutant (**Figure 4B**), indicating that phenazine was indeed inducing production of H_2_O_2_, which then induced catalase (essentially a private good) in the QS-proficient cells but not in the *lasR rhlR* mutant. This increase in catalase activity likely allowed PA14 to detoxify H_2_O_2_ faster than the *lasR rhlR* mutant.

### Mathematical Model of the Competitions

In order to further explore the above-described phenomena, two mathematical models were created: one based on ordinary differential equations (ODEs) and an agent-based model (ABM). The use of ODEs allowed us to verify that the most essential mechanisms of the system had been taken into account as the model could be fitted well to the observed data (Supplementary Materials and Supplementary Figure SM1). Furthermore, the fitting procedure provided quantitative information about several of the parameters involved, such as production rates. Therefore, the same mechanisms were taken into account to set up an ABM.

An ABM is a mathematical spatial model which allows one to simulate the spatio-temporal dynamics of agents (in this case bacterial cells) under diverse conditions. It is composed of an initial spatial configuration of agents and allows simulating the interactions of these agents to assess the collective behavior over time and space. This type of model allows to re-create observed behavior but also to predict the appearance of emergent interactions. Our model was composed of the agents, behavioral and interaction rules as well as an environment (a 2D domain in our case) where all takes place. As an ABM allows us to simulate specific scenarios, this results on numerical simulations, the output of such models. In Supplementary Figure SM2, the result of one of such simulations at a fixed time is shown, the output is visualized as a set of microcolonies of wild-type and QS deficient mutants that grow (in two dimensions) by the assimilation of digestible nutrients (peptides and amino acids), derived from non-digestible ones (caseinate) by the action of exoproteases produced only by the wild-type population. In Supplementary Figure SM3, the dynamics of both populations as a function of time in the absence and presence of pyocyanin (which exerts a bactericidal effect only in the QS deficient mutants) are simulated, showing indeed a decrease in the mutant population as an effect of pyocyanin. Furthermore, the model (i) predicts that pyocyanin would have only very subtle effects on the concentrations of autoinducers, caseinate and exoproteases (Supplementary Figure SM4), (ii) shows how pyocyanin concentration changes with time (Supplementary Figure SM5), and (iii) predicts that an increase of the diffusion of the digestible nutrients strongly increases the selection of the QS deficient mutants.

This type of model can help to choose suitable arrangements for future experiments, to explore certain effects visibly and to understand better the behavior of such systems in a more realistic, heterogeneous environment such as biofilms. Evidence suggests that physical properties of the environment may play an important role in competition (Mund et al., 2017).

### Pyocyanin Restricts the Appearance of Natural Selected Social Cheaters

**FIGURE 5:**
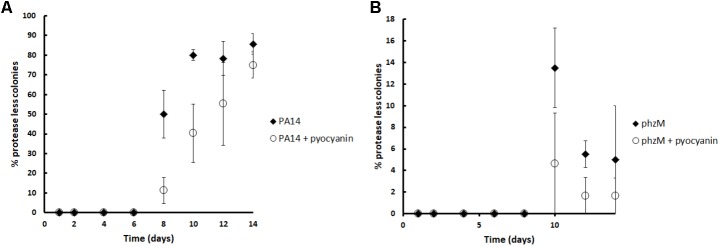
Pyocyanin addition decreases the protease-less individuals (social cheaters) in casein cultures. Experiments were done in triplicate, and the average is shown. Pyocyanin (100 μM) was added to the medium before each daily inoculation, and subsequent cultures were done every 24 h. Experiments were done in triplicate, and the average ± SEM is shown. The difference between the control and the pyocyanin curve was significant at 8, 10, and 14 days according a Chi-squared test (*P* < 0.05).

In addition to the short time competition experiments, we decided to test if pyocyanin could restrict the appearance of social cheaters in long term experiments in a population that was entirely composed of wild-type individuals at the beginning. For this, cultures in M9 caseinate medium were started from a single wild-type colony and several consecutive daily subcultures in the same medium were done with or without the addition of 100 μM of pyocyanin before each pass. As expected, after eight culture passes, protease-less mutants (cheaters) began to appear and their proportion increased until pass number fourteen; however, in agreement with our previous experiments, the addition of pyocyanin significantly decreased the proportion of social cheaters at days 8, 12, and 14 (**Figure 5A**). As expected, pyocyanin addition also decreased the cheater proportion in cultures initiated with the *phzM* mutant (**Figure 5B**); nevertheless, these differences were not significant and the proportion of cheaters that emerged in the *phzM* background was lower than the observed for the wild-type strain.

### Pyocyanin Selects Active QS in *A. baumannii*

**FIGURE 6:**
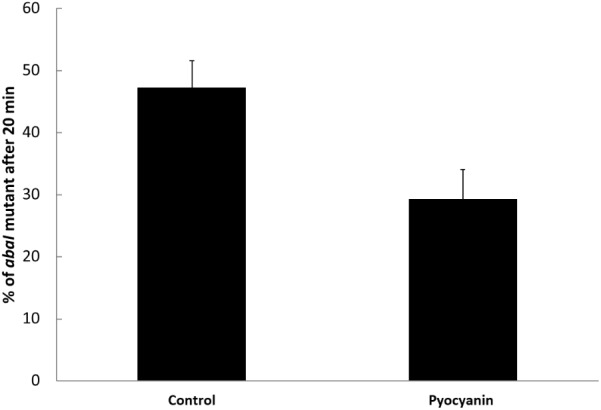
Pyocyanin selects the wild-type QS+ phenotype in *Acinetobacter baumannii*. Experiments were done in quintuplicate and the average ± SEM is shown. The difference was significant according a two tiled *t*-test (*P* < 0.05).

After demonstrating that pyocyanin selected for active QS in *P. aeruginosa*, we also tested its effect in another bacterial species, *A. baumannii*, an important nosocomial pathogen able to coexist with *P. aeruginosa* and a strain that upregulates its catalase activity via QS. It was previously shown that the deletion of *abaI* impairs production of homoserine lactone and increases sensitivity to pyocyanin (Bhargava et al., 2014). To test if pyocyanin could also select QS in *A. baumannii*, competitions between the wild-type ATCC17978 strain and its isogenic *abaI* mutant were conducted by mixing equal quantities of both strains grown in LB (in which they grow with virtually identical growth rates of 1 ± 0.05 and 1.03 ± 0.01 h^−1^, Supplementary Figures S2A,C) and growing to the exponential phase and by incubating for 20 min with 50 μM of pyocyanin. These competitions were made only at short times to avoid sensing of the wild-type-produced homoserine lactone by the *abaI* mutant. In order to estimate the initial and final proportions of the strains, PCR amplification of the *abaI* gene was performed. Our results showed that without pyocyanin, the initial mutant proportion remained stable, whereas the addition of pyocyanin decreased the mutant to around half the initial proportion (**Figure 6**). These results, as for *P. aeruginosa*, correlate with higher catalase activity of the wild-type strain relative to the activity of the QS defective mutant and with a significant induction of catalase by the addition of pyocyanin only in the case of the wild-type strain (Supplementary Figure S3). Hence, we found that the pyocyanin produced by *P. aeruginosa* can select for QS in another bacterial system; therefore, the influence of pyocyanin in bacterial ecology may be broad.

## Discussion

In this work, we demonstrated that pyocyanin is able to select functional QS systems in both *P. aeruginosa* and *A. baumannii*. The toxicity of pyocyanin is due its generation of oxygen-reactive species, including H_2_O_2_, that cause cell lysis (Das and Manefield, 2012), Our results are in concordance with the fact that in *P. aeruginosa*, QS mutants are more sensitive to several kinds of stress than the strains with functional QS systems (García-Contreras et al., 2015b). These stresses include heat shock, heavy metal stress, and oxidative stress by H_2_O_2_ (García-Contreras et al., 2015b). Hence, the addition of H_2_O_2_ selects for active QS systems during growth on casein as the sole carbon source, which requires the production of QS-controlled exoproteases (García-Contreras et al., 2015b). The lower tolerance of QS mutants to stress may be linked to a deficient expression of antioxidant enzymes during an insult (García-Contreras et al., 2015b), as well to other phenomena such as the production of less robust cell membranes by the QS deficient individuals (Davenport et al., 2015). Critically, stress in general is omnipresent in the variable natural environments of bacteria, and oxidative stress in particular is a common strategy used to fight bacterial pathogens in eukaryotes (Hampton et al., 1998; Abramovitch and Martin, 2004; Vallet-Gely et al., 2008).

Interestingly, a recent publication in which the role of nitrogen source in the selection of social cheaters was evaluated; it shows that in those cultures in which the population remained stable (tragedy of the commons was avoided), the cooperators evolved by decreasing their exoprotease production and by increasing their production of pyocyanin (Lai et al., 2018). This result complements our findings by suggesting that overexpression of this toxin may be a natural strategy to counteract social cheating.

Although for this work we did not explore the effect of pyocyanin in competitions under conditions in which cheating is not present, pyocyanin may also select the wild-type phenotype under these kinds of conditions over QS defective mutants. Moreover, other redox active phenazines like phenazine-1-carboxylic acid may also participate in QS selection, but this remains to be explored. In addition, there may be possible contributions to the observed effects mediated by the pyocyanin biosynthetic intermediate 5-methyl phenazine-1-carboxylic acid since the *phzM* mutant also is deficient in its production.

Intriguingly, although we predicted that a higher number of cheaters would arise in the case of *phzM* cultures than in the wild-type cultures (since the wild-type strain produces pyocyanin), we observed the opposite (**Figure 5B**). We speculate that this result may be caused by a lower exoprotease activity of the *phzM* mutant compare to the parental strain. Accordingly, the *phzM* mutant has significantly lower collagenase and elastase activities (data not shown) which will decrease cheater fitness. In addition, we cannot rule out the presence of compensatory mechanisms that counteract the cheater fitness, like perhaps a higher synthesis of HCN. Nevertheless, this hypothesis needs to be tested.

Generally, various kinds of stress tend to be positively correlated with cell density due to competition for resources or the accumulation of waste products. This probably explains why an increasing number of private stress responses are shown to be QS regulated (Cornforth and Foster, 2013; García-Contreras et al., 2015b). We thus hypothesize that – as a side effect – one of the main stabilizing mechanisms of cooperation via public goods are QS-regulated private stress responses (García-Contreras et al., 2015b), although QS controlled stress responses do not need to be private (Friman et al., 2013; Sun et al., 2015).

Stabilization of cooperation by the QS-regulated stress response acts complementarily to stabilization by metabolic prudence (Xavier et al., 2011). The metabolic prudence concept explains that cells produce public goods preferentially when the cost of their production and impact on individual fitness is low, and under such conditions non-producing cheaters have no fitness benefit. In contrast, our study showed that pleiotropic stabilization of cooperation via public goods also works when cooperative production of public goods is associated with fitness costs for the producer.

Overall, we demonstrate that although cheating decreases the fitness of QS proficient individuals in the absence of stress, the higher susceptibility of QS mutants to the pyocyanin produced by the wild-type strain allows for the preservation of functional QS systems; i.e., pyocyanin promotes the selection of more virulent bacteria. Hence, our results provide a new example of social policing by the cooperators and complement the findings of Wang and colleagues that in 2015 showed that QS-controlled production of toxic HCN by cooperative individuals decreases the fitness of the social cheaters, because social cheaters are more susceptible to HCN than cooperators, presumably by the lack of expression of an HCN insensitive cytochrome oxidase that is positively controlled by QS (Wang et al., 2015).

## Author Contributions

PC-T, JR-P, MS-M, YG, and MT performed the experiments. JP-V, CK, and AJ made the mathematical model. RG-C, JP-V, CK, TM, JJ-C, and TW conceived the study. RG-C, JP-V, CK, and TW wrote the manuscript. JJ-C analyzed the data.

## Conflict of Interest Statement

The authors declare that the research was conducted in the absence of any commercial or financial relationships that could be construed as a potential conflict of interest.
