# Supplementary material for: Pyocyanin Restricts Social Cheating in Pseudomonas aeruginosa
Source: Front Microbiol. 2018 Jun 27;9:1348. doi: 10.3389/fmicb.2018.01348 (PMC6030374; doi:10.3389/fmicb.2018.01348)
Supplement: Supplementary file 1 [file Table_1.docx]

Supplementary material

| **PCR** **KNOCKOUT and SEQUENCING** | | | |
| --- | --- | --- | --- |
|  | **Primer Sequence (5´-3´)** | | |
| AbaIUp(FPstI)  AbaIUp(EcoRI) | Fow  Rev | GCGCTGCAGCGCGAGTAGTAGCAATATTGAC  CCCGAATTCGGGATCAAATTGATCTAGTT | |
| AbaIDown(EcoRI)  AbaIDown(BamHI) | Fow  Rev | GGGGAATTCCCCTCATTACGACTTCTCCTC  GCGGGATCGCGTCATCCAAAGCAACTCATCACC | |
| AbaI-Int | Fow  Rev | GAGAATCCAATATCATTGGTTGTGCC  GCGCCTTGTTCTCTTGCAAAGTTG | |
| Plasmid pMo130 | Fow  Rev | ATTCATGACCGTGCTGAC  CTTGTCTGTAAGCGGATG | |
| **qRT-PCR** | | | |
|  | **Primer Sequence (5´-3´)** | | **Taqman Probe** |
| AbaI^a^ | Fow  Rev | CCGCTACAGGGTATTTGTTGAAT  CTTGATAATTCCCAAATTTCTGG | 6FAM-CGATACAGCCTATGTCGTGGCTCAAG  -BBQ |

^a.^ AUO97_06645: Number accession sequence of *abaI* gene from *A.baumannii* ATCC 17978
