# Supplementary material for: Pyocyanin Restricts Social Cheating in Pseudomonas aeruginosa
Source: Front Microbiol. 2018 Jun 27;9:1348. doi: 10.3389/fmicb.2018.01348 (PMC6030374; doi:10.3389/fmicb.2018.01348)
Supplement: Supplementary file 2 [file Presentation_1.PPTX]

## Slide 1
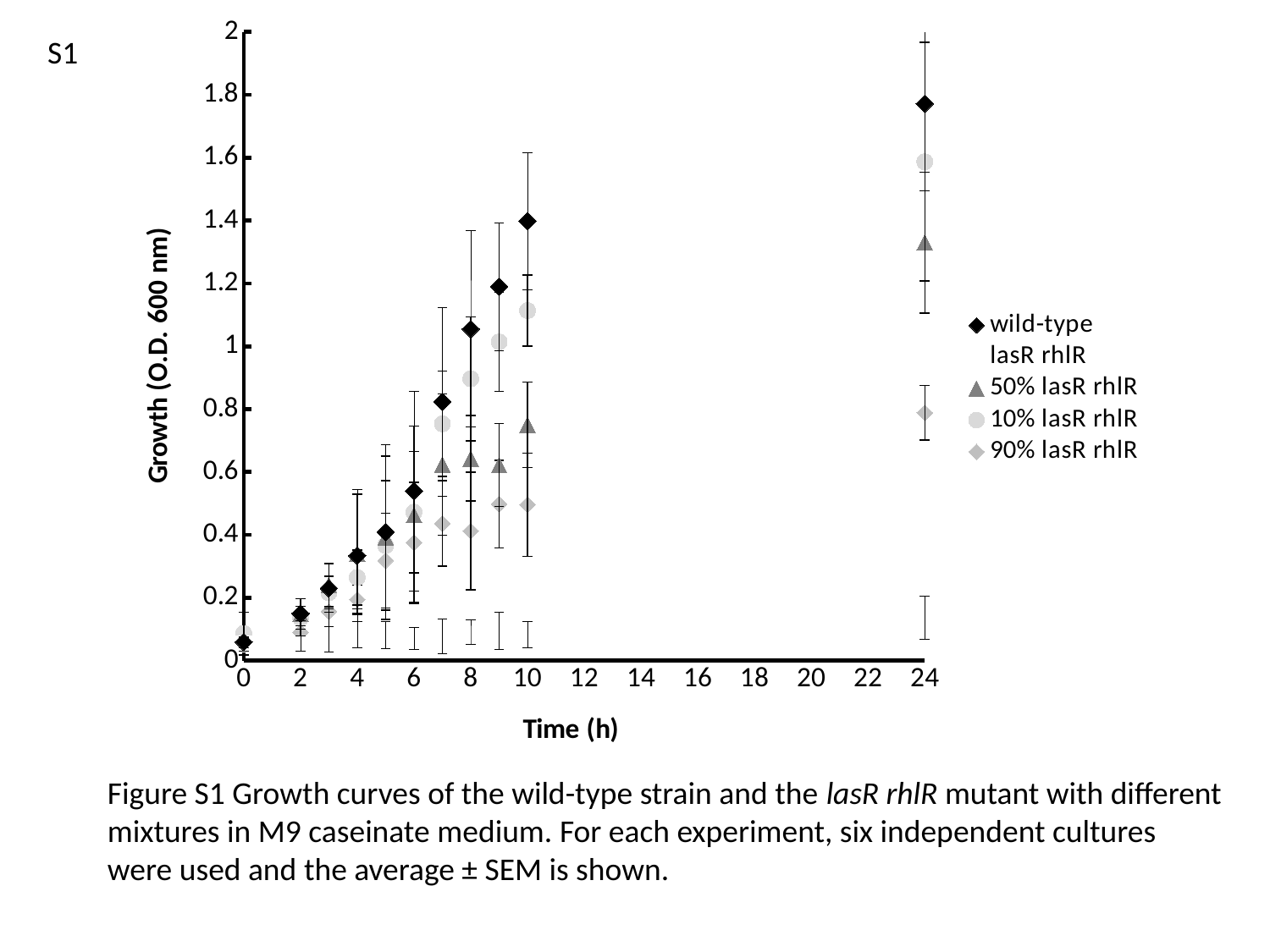

### Chart
| Category | wild-type | lasR rhlR | 50% lasR rhlR | 10% lasR rhlR | 90% lasR rhlR |
|---|---|---|---|---|---|S1
Figure S1 Growth curves of the wild-type strain and the lasR rhlR mutant with different mixtures in M9 caseinate medium. For each experiment, six independent cultures were used and the average ± SEM is shown.

## Slide 2
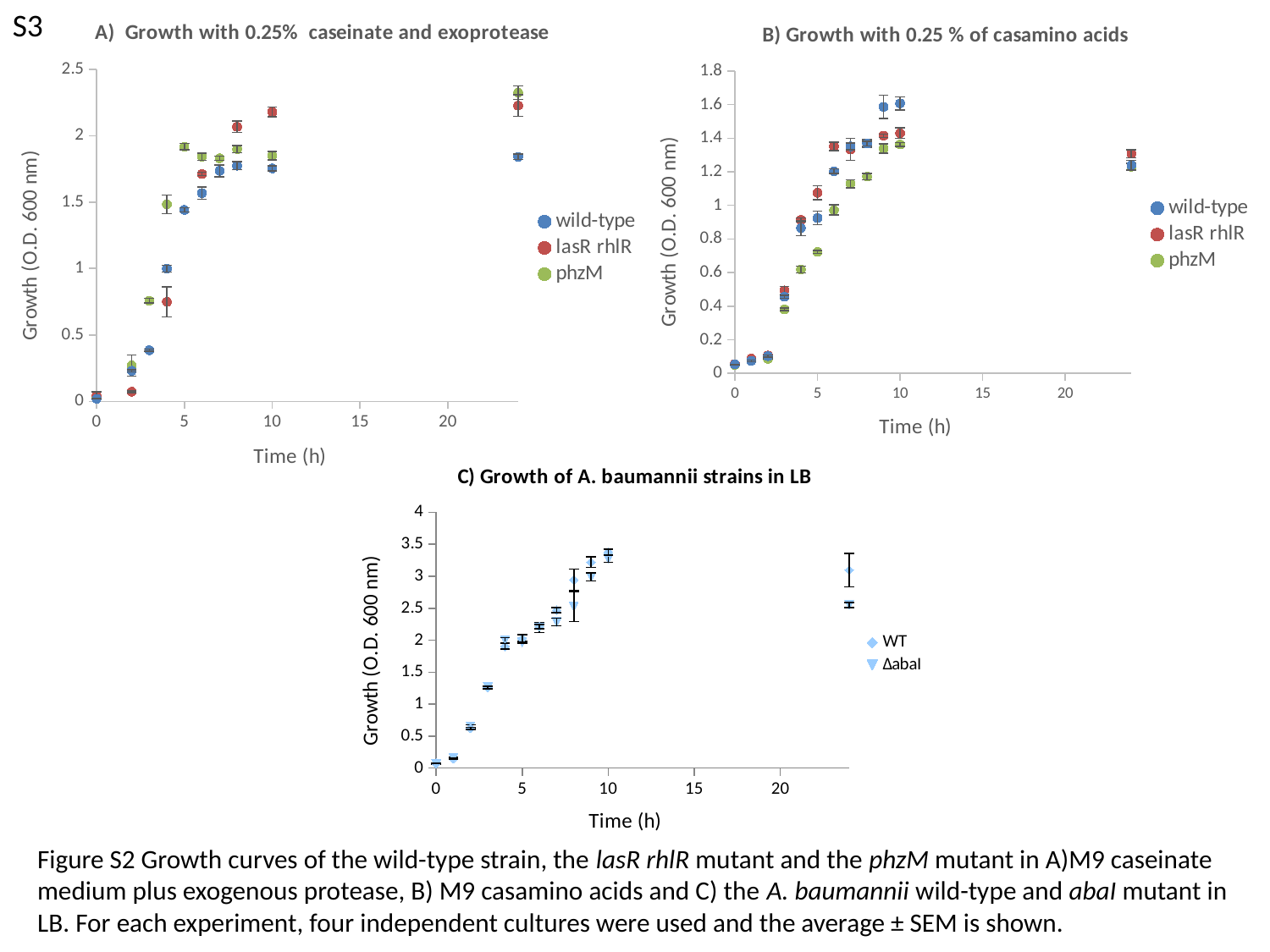

S3
### Chart: A) Growth with 0.25% caseinate and exoprotease
| Category | wild-type | lasR rhlR | phzM |
|---|---|---|---|
### Chart: B) Growth with 0.25 % of casamino acids
| Category | wild-type | lasR rhlR | phzM |
|---|---|---|---|
### Chart: C) Growth of A. baumannii strains in LB
| Category | WT | ΔabaI |
|---|---|---|Figure S2 Growth curves of the wild-type strain, the lasR rhlR mutant and the phzM mutant in A)M9 caseinate medium plus exogenous protease, B) M9 casamino acids and C) the A. baumannii wild-type and abaI mutant in LB. For each experiment, four independent cultures were used and the average ± SEM is shown.

## Slide 3
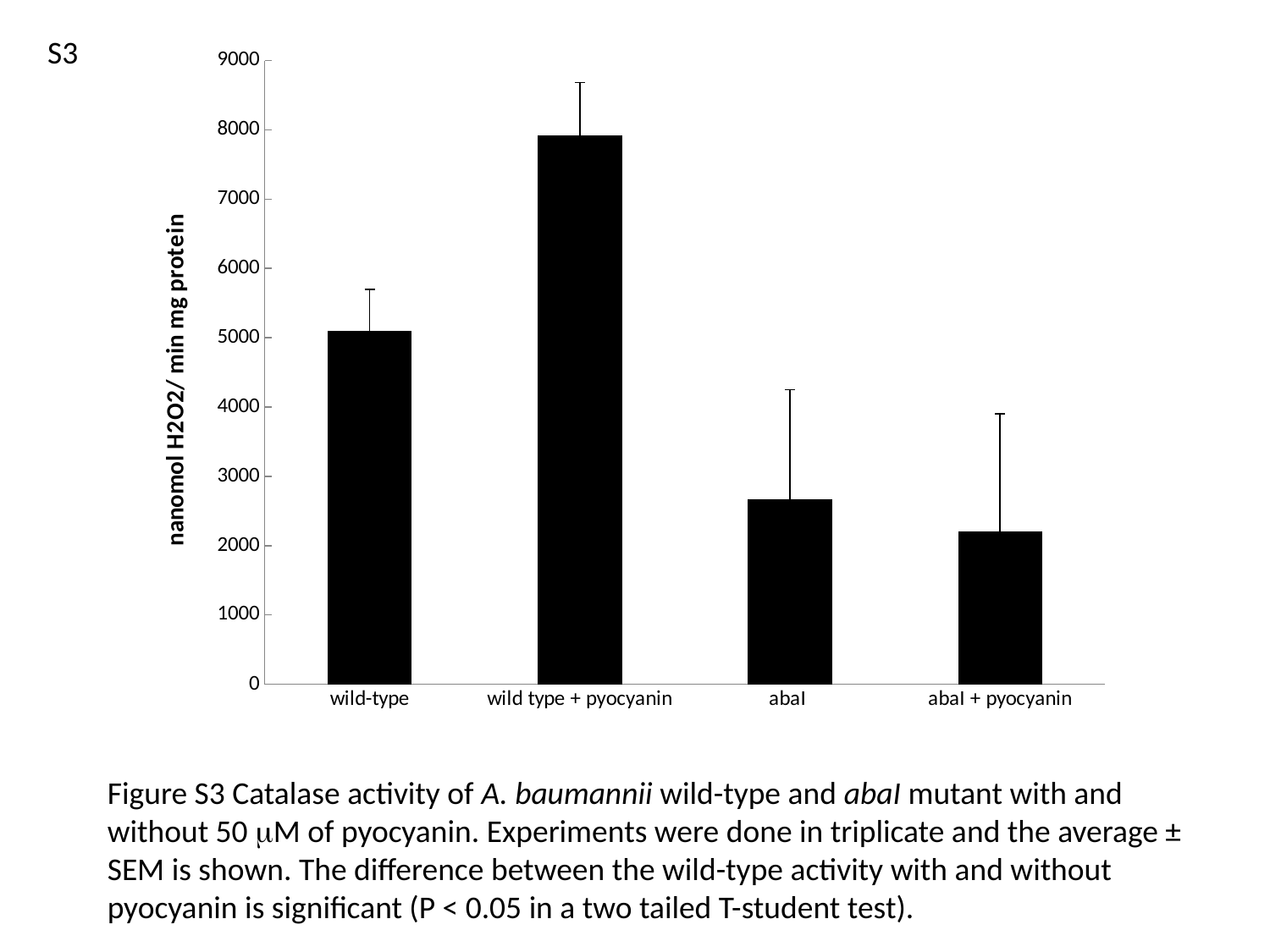

S3
### Chart
| Category | |
|---|---|
| wild-type | 5099.8502899055675 |
| wild type + pyocyanin | 7916.858502389972 |
| abaI | 2667.524554663332 |
| abaI + pyocyanin | 2203.4732102804596 |Figure S3 Catalase activity of A. baumannii wild-type and abaI mutant with and without 50 mM of pyocyanin. Experiments were done in triplicate and the average ± SEM is shown. The difference between the wild-type activity with and without pyocyanin is significant (P < 0.05 in a two tailed T-student test).
