# Supplementary material for: Pyocyanin Restricts Social Cheating in Pseudomonas aeruginosa
Source: Front Microbiol. 2018 Jun 27;9:1348. doi: 10.3389/fmicb.2018.01348 (PMC6030374; doi:10.3389/fmicb.2018.01348)
Supplement: Supplementary file 3 [file Data_Sheet_1.PDF]

Supplementary material

The experiments presented in the main text indicated that QS-regulated pyocyanin production by the wild-type population in *Pseudomonas aeruginosa* could work as a policing mechanism to select functional QS systems. Following the experiments, we developed two mathematical models to describe how pyocyanin concentration may impact on the evolutionary fitness of mixed bacteria populations of *P. aeruginosa* wildtype (PA14) and the *lasRrhlR* mutant.

The first model is composed of differential equations (deterministic) to describe the QS dynamics, and the second model is an agent based model to explore how environment diffusion properties and non-homogeneous distribution of bacteria may play a role in stabilizing QS. In [2], we provide detailed information regarding the model development and therefore, here, we only provide a brief description.

The hybrid model is composed of a differential equations (deterministic) model coupled with an agent based model. The differential equations model consists of ordinary differential equations (ODE) to describe the QS system and qualitatively reproduce the experimental dynamics. The aim here was to describe the QS and population dynamics. This part of the model served to check for the most essential mechanisms and to obtain a rough estimation for the parameters values involved. We use this information later to feed the spatial model. Note that a specific representation of each interaction step at the molecular level was not the object of this model, as this indeed it is not possible based on the actual state of knowledge.

1 The ordinary differential equations (ODE) model

To build the ODE model, we focused on the following assumptions:

- Homogeneously mixed populations, i.e. no diffusion or other spatial effects.
- Competition between wildtype B_w and double mutant B_c: both consume the same digestible limited nutrient N.
- Both population types have distinct basic growth rates.
- Standard QS model only for Las-System in *P. aeruginosa*, with positive feedback loop, $AHL = A$.
- Exoenzymes E (QS-regulated) can degrade non-digestible nutrients ($\tilde{N}$) into digestible ones (N), i.e. QS induced production of public goods provides benefit to both wildtype and cheater cells.
- Pyocyanin P production is also under QS-control.
- Most enzymatic transformations and nutrient consumption terms etc. are assumed to be limited (Michaelis-Menten-like).

ODE Model equations

$$\frac{dB_w}{dt} = r_w \frac{N}{K_w + N} B_w - d_w B_w \tag{1a}$$

$$\frac{dB_c}{dt} = r_c \frac{N}{K_c + N} B_c - d_c B_c - \xi_1 \frac{P}{K_P + P} B_c \tag{1b}$$

$$\frac{dA}{dt} = \alpha + \beta \frac{A^n}{C_1^n + A^n} B_w - \gamma_A A \tag{1c}$$

$$\frac{dP}{dt} = \beta_P \frac{A^n}{C_1^n + A^n} B_w - \gamma_P P - \xi_2 P \frac{1}{K_P + P} B_c \tag{1d}$$

$$\frac{d\tilde{N}}{dt} = -c_n E \frac{\tilde{N}}{\tilde{N} + K_{\tilde{N}}} - \gamma_{\tilde{N}} \tilde{N} \tag{1e}$$

$$\frac{dN}{dt} = c_n E \frac{\tilde{N}}{\tilde{N} + K_{\tilde{N}}} - \frac{1}{Y_W} r_w \frac{N}{K_w + N} B_w - \frac{1}{YC} r_c \frac{N}{K_c + N} B_c - \gamma_n N \tag{1f}$$

$$\frac{dE}{dt} = \beta_E B_w \frac{A^n}{C_1^n + A^n} - \gamma_E E \tag{1g}$$

The associated parameters can be found in table SM0, where we denote in grey cells parameters concerning the basic QS/regulation system.

r_w wild-type growth rate	α basic AHL production
r_c cheater growth rate	β activated AHL production
d_w wild-type death rate	γ_A abiotic AHL degradation rate
d_c cheater death rate	n Hill coefficient
K_w half max. for growth wildtype	C_1 AHL threshold
K_c half max. for growth cheater	β_P Production of pyocyanin by bacteria
ξ_1 max. killing rate by pyocyanin	ξ_2 consumption of pyocyanin by killing bacteria
K_P Pyocyanin concentration for half max killing rate	γ_P abiotic pyocyanin degradation
γ_E abiotic decay of exoenzymes	c_n transformation rate into digestible nutrients by exoenzyme E
$K_{\tilde{N}}$ half max non-digestible nutrients concentration	$\gamma_{\tilde{N}}$ abiotic decay/disappearance of non-digestible nutrients
γ_n abiotic decay / disappearance of digestible nutrients	Y_W yield rate for wildtype
Y_C yield rate for cheater	β_E production rate for exoenzymes

Table SM0: ODE model parameters. Parameters in grey cells are QS-related parameters.

1.1 Numerical solutions and results

We solve system (1) numerically using the appropriate initial conditions for each competition case: control case, 10, 50 and 90 % initial cheater population. We chose parameters reflecting the qualitative behaviour of the competition experiments. For more details, see [2].

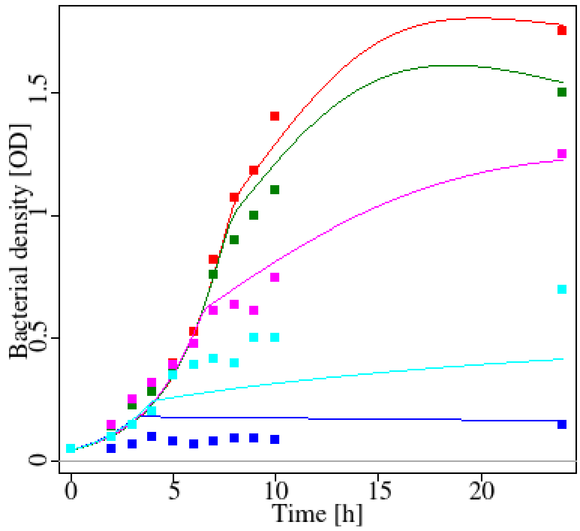

Figure SM1: ODE simulations (solid lines) for the time course of the total bacterial density fitted to experimental data (squares), in different initial proportions of the QS-mutant: 0% (red), 10% (green), 50% (magenta), 90% (cyan), 100% (blue).

2 The agent based model (ABM)

The main goal of this model is to investigate spatially the effects of pyocyanin on competition experiments between *P. aureginosa* wild-type and QS deficient l*asR rhlR* mutant strains. The model is two-dimensional and developed using the NetLogo simulation platform. The model is described based on ODD protocol [1]. For some background information and more details about this modeling approach, see [2] - we follow the procedure there.

The main idea for the spatially structured ABM is to identify the agents with the bacterial cells. Here, we have two types of agents included: wild types and QS deficient mutants. They are located in patches and acting independent of each other, but of course reacting on and producing exoenzymes, AHL, nutrients and pyocyanin. The model environment consists of a surface of 100 μm $\times 100\mu m$, with a constant height of $10\mu m$. The size of bacteria is approximately 1 μm. The time steps for the simulations are chosen to be one minute. The variables are described in Table SM1. The dynamics of the system is defined recursively (using the state at time step t to calculate the state at time step $t + 1$).

Variable	Unit	Initial Value	Description
PG	nM	8	Concentration of digestible nutrient in the patch
Exo	nM	2	Concentration of exoenzyme in the patch
NDN	nM	204	Concentration of non-digestible nutrient in the patch
Bw	-	10,50,90	Number of Wild-type bacteria in the patch
Bc	-	10,50,90	Number of QS deficient mutant bacteria in the patch
AHL	nM	70.3	Concentration of AHL in the patch
Pyo	nM	0	Concentration of pyocyanin in the patch
BcDR	nM	0	QS deficient mutants death rate in presence of pyocyanin

Table SM1: ABM model initial values of substances at the beginning of the simulation.

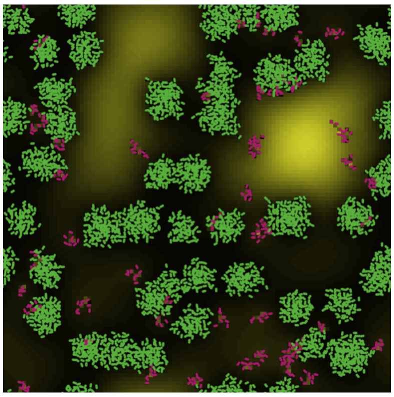

Figure SM2: Exemplary snapshot (at a fixed time) of a simulation of the ABM. Wild type cells are shown in green and cheaters in magenta.

The model also allows to track dynamically (changes in time) of several of the concentrations which are relevant, such as AHL, nutrients and exo-enzymes. Particularly, it allows us to see directly the quantitative effect that adding pyocyanin has on these concentrations. Additionally, we can track how pyocyanin dynamically changes with time. See figures SM4 and SM5, respectively.

2.1 Effects of diffusion

Given that a spatial model allows to further explore the contemplated scenarios, we use it to investigate whether physical properties of the environment in which the competition takes place (such as diffusion) plays a further role in the competition outcome. This was not explored experimentally but numerically with the ABM. We found evidence pointing towards diffusion properties actively affecting the competition, in particular high diffusion rates may facilitate mutant invasion, given that access to public goods is faster. See figure SM6. This observation may have an impact where different diffusion rates are found in different growth conditions (for example in patients with and without cystic fibrosis).

References

[1] Volker Grimm, Uta Berger, Donald L. DeAngelis, J. Gary Polhill, Jarl Giske, and Steven F. Railsback. The odd protocol: A review and first update. *Ecological Modelling*, 221(23):2760–2768, 11 2010.

[2] Ammar Jalalimanesh, Christina Kuttler, Rodolfo García-Contreras, and Judith Pérez-Velázquez. *An Agent-Based Model to Study Selection of Pseudomonas aeruginosa Quorum Sensing by Pyocyanin: A Multidisciplinary Perspective on Bacterial Communication*, pages 133–147. Springer International Publishing, Cham, 2018.

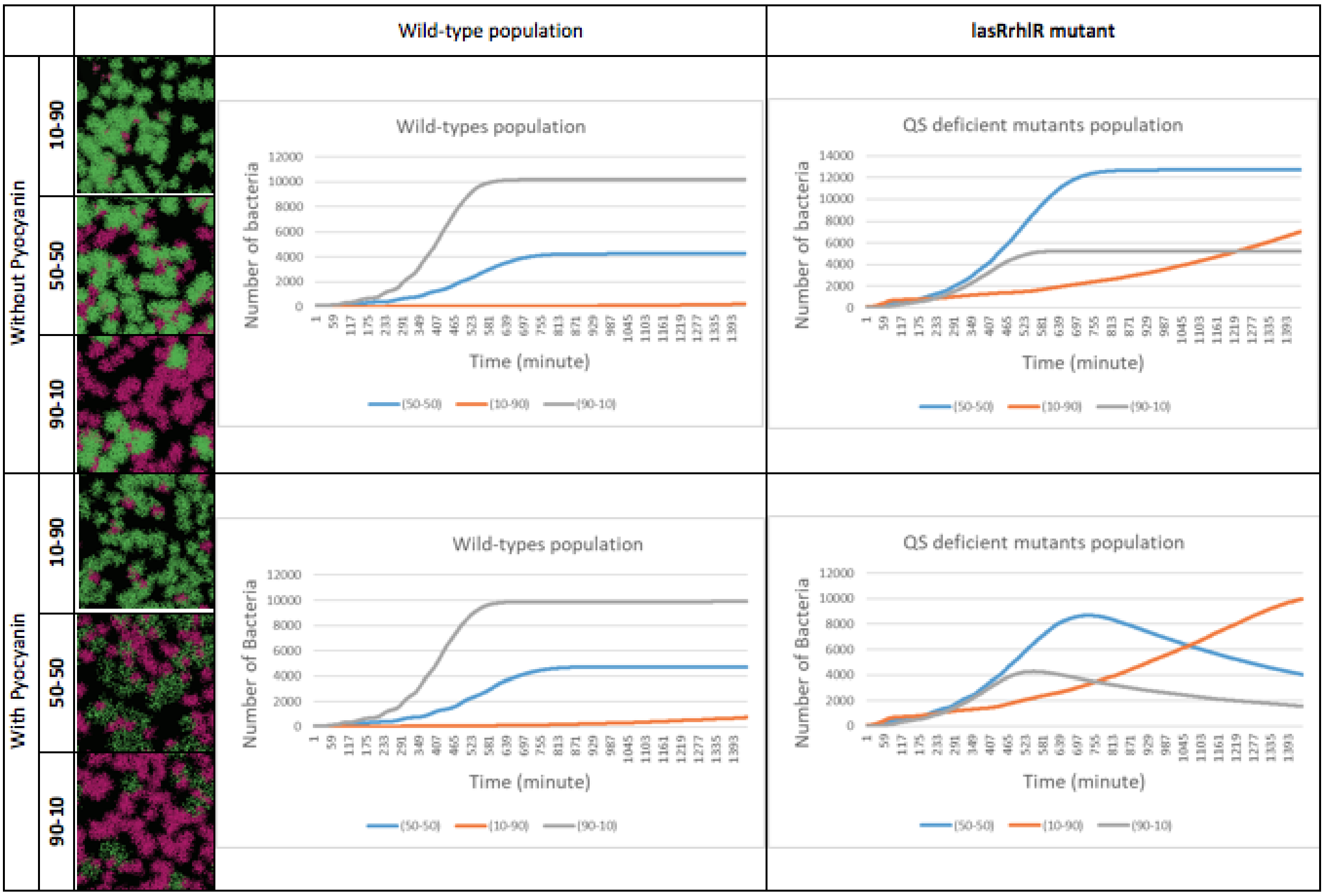

Figure SM3: Effects of pyocyanin on bacterial growth for all three contemplated scenarios (10, 50 and 90 initial percentage of *lasRrhlR* mutant). The snapshot of the simulation are at the end of the simulation.

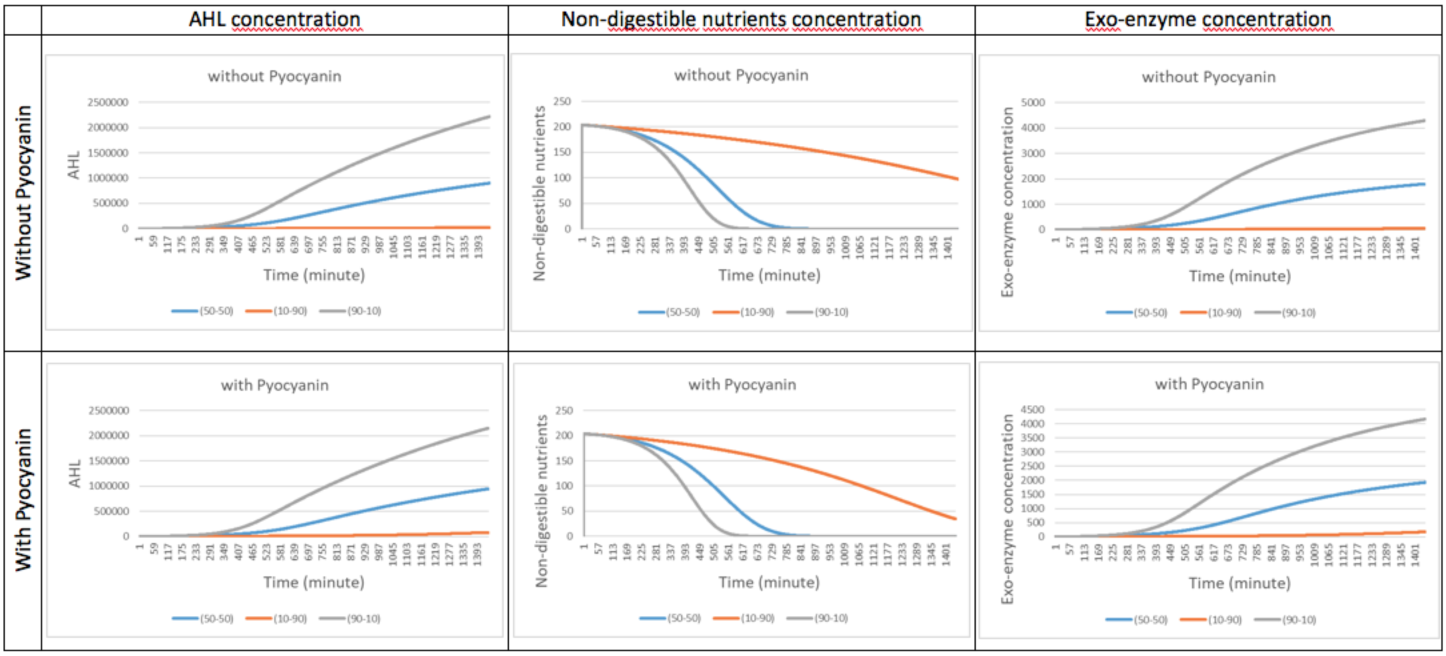

Figure SM4: How pyocyanin affects AHL, non-digestible nutrients and exo-enzyme concentration with time.

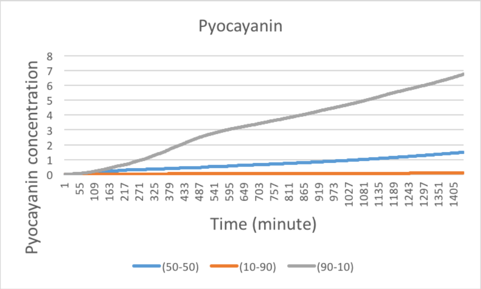

Figure SM5: Pyocyanin dynamics.

	Population growth	Proportion of the population
Diffusion update times per minute (N) = 10 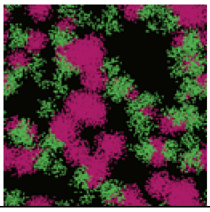	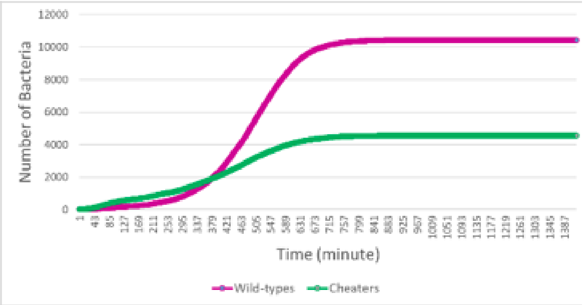	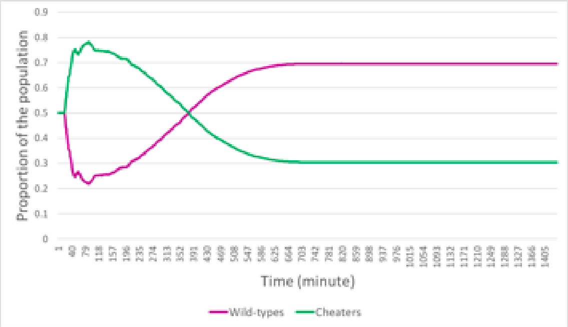
Diffusion update times per minute (N) = 100 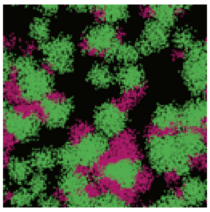	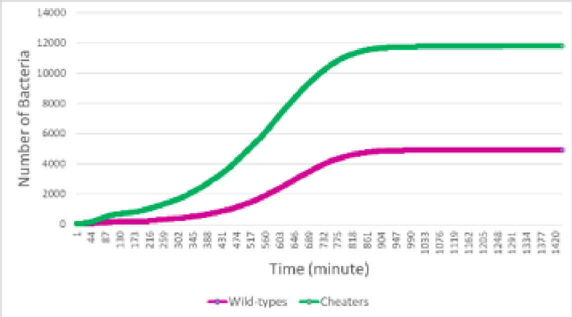	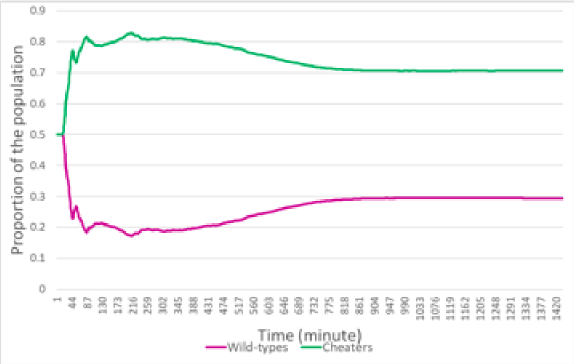

Figure SM6: Simulation of the effects of diffusion on population growth.
